# Health-related quality of life in patients with knee osteoarthritis attending two primary care clinics in Malaysia: a cross-sectional study

**DOI:** 10.1186/1447-056X-8-10

**Published:** 2009-12-31

**Authors:** Zainal F Zakaria, Azman A Bakar, Hadzri M Hasmoni, Fauzi A Rani, Samiah A Kadir

**Affiliations:** 1 Putrajaya Health Clinic, Presint 9, 62250 Putrajaya, Malaysia; 2 Institute for Health Systems Research, Ministry of Health Malaysia, Jalan Rumah Sakit Bangsar, 59000 Kuala Lumpur, Malaysia; 3 Department of Medicine, International Islamic University Malaysia, Jalan Istana, Bandar Indera Mahkota, 25200 Kuantan, Pahang Darul Makmur, Malaysia; 4 Department of Family Medicine, Faculty of Medicine, Universiti Kebangsaan Malaysia Medical Centre, Jalan Yaakob Latif, Cheras, 56100 Kuala Lumpur, Malaysia

## Abstract

**Background:**

Measurement of health-related quality of life (HRQOL) among patients with osteoarthritis (OA) helps the health care provider to understand the impact of the disease in the patients' own perspective and make health services more patient-centered. The main aim of this study was to measure the quality of life among patients with symptomatic knee OA attending primary care clinic. We also aimed to ascertain the association between socio-demographic and medical status of patients with knee OA and their quality of life.

**Methods:**

A clinic based, cross sectional study using the Short Form-36 (SF-36) questionnaire was conducted in two primary care health clinics in Hulu Langat, Selangor, Malaysia over a period of 8 months. The nurses and medical assistants were involved in recruiting the patients while the family physicians conducted the interview.

**Results:**

A total 151 respondents were recruited. The mean age was 65.6 ± 10.8 years with females constituted 119 (78.8%) of the patients. The mean duration of knee pain was 4.07 ± 2.96 years. Half of the patients were overweight and majority, 138 (91.4%), had at least one co-morbidity, the commonest being hypertension. The physical health status showed lower score as compared to mental health component. The domain concerning mental health components showed positive correlation with age. There was a significant negative correlation between age and physical functioning (p < 0.0005) which indicated the deterioration of this domain as patients became older. Male respondents had better scores in most of the QOL dimensions especially in the physical functioning domain (p = 0.03). There was no significant association between QOL with different education levels, employment status and marital status. Patients with higher body mass index (BMI) and existence co-morbidities scored lower in most of the QOL domains.

**Conclusions:**

This study has shown that patients with knee OA attending primary care clinics have relatively poor quality of life pertaining to the physical health components but less impact was seen on the patients' mental health.

## Background

Health-related quality of life (HRQOL) is increasingly acknowledged as a valid health indicator in many diseases. HRQOL is narrowed to aspects of an individual's life that is affected by health, disease and/or its treatment. It encompasses emotional, physical, social and subjective feelings of well being that reflect an individual's subjective evaluation and reaction to his/her illness [1].

Osteoarthritis (OA) is the most common type of arthritis found worldwide especially in the elderly. It is a major cause of disability in both the developed and developing world [2]. The prevalence of OA is in the region of 10-20% of the adult population. In all populations studied so far the prevalence of knee OA is higher than that of hip OA but this is more marked in Asian populations [3]. The most common form of OA in Malaysia is knee OA [4]. Although there is no exact figure of patients with knee OA, the Community Orientated Program for Control of Rheumatic Diseases (COPCORD) study showed that 9.3% of adult Malaysians complained of knee pain with a sharp increase in pain rate to 23% in those over 55 years of age and 39% in those over 65 years [5,6].

Most patients with OA are assessed and treated within primary care settings [7] but there seems to be a discrepancy between how doctors and patient define the importance of an illness. Furthermore, OA of the knee is often ignored by doctors until the disease is very advanced because it is often considered as part of the 'normal' ageing process [7,8]. As OA and other rheumatic conditions seldom cause death but have a substantial impact on health, HRQOL measures are better indicators of their impact than related mortality rates [9].

Review of medical literature revealed 15 studies on HRQOL in OA but there were only two studies that specifically looked at HRQOL in patients with OA in the primary care setting [10,11]. HRQOL studies in patients with knee OA attending primary care have never been done in Malaysia. Our study specifically aims to look at the HRQOL of patients with symptomatic knee OA in primary care setting. The findings will help the care provider especially in the primary care to understand the impact of the disease on the patients and offer an insight into the need for early treatment and interventions.

We documented the socio-demographic, medical and non-medical related characteristics of these patients and ascertained their association with HRQOL. We have found that patient with knee OA attending primary care have relatively poor quality of life pertaining to the physical health components but there was less impact on the mental health of the patients.

## Methods

This was a cross-sectional study involving patients with symptomatic knee OA attending two different health clinics in Hulu Langat, Selangor, Malaysia. The study was conducted between 1st September 2003 and 30th April 2004. By universal sampling, all patients aged 50 and above visiting the outpatient clinic were screened by a triage nurse or medical assistant for knee pain. Their names and registration numbers were recorded in a special registry book. If the patients were not seen on the same day, they were seen at the next visit. All patients diagnosed with knee OA based upon the American College of Rheumatology criteria [12] and age 50 years and above were included. We excluded those who were illiterate and could not answer the questionnaire. Patients who needed hospital admission or those with any other forms of lower limb immobility or abnormality such as paraplegia were also excluded.

The medical outcome study 36-item short form (SF-36) was used to measure the HRQOL in this study. The SF-36 is a 36-item instrument designed to measure generic health concepts relevant across age, disease and treatment groups. It is a reliable and validated generic instrument that has been used extensively to measure HRQOL in diverse groups [13]. Through the International Quality of Life assessment Project, 15 countries including Malaysia have participated in translating and adapting the SF-36.

Socio-demographic and medical characteristics of the patients were recorded and the Malay language version of SF-36 form was either self-administered or led by an interviewer (face-to-face). This version had been translated and validated and was used in the Malaysian National Quality of Life Survey 2000 [14]. It measured eight domains consisted of physical functioning, role-physical, bodily pain, general health, vitality/energy, social functioning, role-emotional and mental health. Scores on each scale ranged from a minimum of 0 to a maximum of 100, with higher scores indicative of better health. Proxy respondents from either family or friends were not entertained as the validity of SF-36 will be compromised. If there was problem in understanding the questionnaire, the investigator would re-read it without re-phrase or explanation. All respondents were asked to answer based on what they understood of the question.

Information for computation of scores was provided by the manual produced by the Medical Outcomes Trust (1994). Statistical Package for Social Sciences, version 12.0 (SPSS Inc, Chicago, IL) was used for data analysis. Age, body mass index and duration of knee pain were entered as continuous variables. Other variables were entered as binary categorical. The association between continuous quantitative variable and QOL scores was analyzed using Pearson Correlation Test. Independent samples T-Test was used to analyzed association between dichotomous qualitative variables and QOL scores. The association of polinomial qualitative variables and QOL scores were analyzed with One-way Analysis of Variance (ANOVA). The p value significance was taken as < 0.05. Ethnicity was analyzed as dichotomous variable (Malay and non-Malay) due to small proportion of other ethnics. Results with showed statistical significance were re-analysed using the multiple linear regression models to control the confounding factors.

## Results

There were a total of 213 patients with knee pain during the study period and only 154 were eligible. Out of this, only 151 (98%) completed the study. The patients' baseline and socio-demographic characteristics are shown in Table 1.

**Table 1:** Patients baseline and socio-demographic characteristics

Patient characteristics	Number of patients, N = 151
**Age, yr (mean ± SD)**	65.6 ± 10.8
**Gender**	
Male	32 (21.2%)
Female	119 (78.8%)
	
**Race**	
Malay	119 (78.8%)
Chinese	16 (10.6%)
Indian	9 (6%)
	
**Marital status**	
Married	114 (75.5%)
Single/Widowed/Divorce	37 (24.5%)
	
**Social support**	
Living alone	5 (3.3%)
Living with relatives	146 (96.7%)
	
**Level of education received**	
No formal education	20 (13.3%)
Primary	100 (66.2%)
Secondary	31 (20.5%)
Tertiary	0 (0%)
	
**Employment status**	
Employed/Self-employed	17 (11.2%)
Unemployed/Retired	134 (88.8%)

Thirteen (8.6%) patients were not known to have any of the surveyed medical illnesses and 4 (2.6%) had more than four. The most common was hypertension and the other medical characteristics are shown in Table 2. The mean duration of knee pain was 4.07 ± 2.96 years and only eight (5.3%) patients had been hospitalized for the disease in the last one year. The mean body mass index (BMI) was 28.23 ± 4.97 with majority (n = 76, 50.3%), were in the overweight category.

**Table 2:** Patients Medical Characteristics

		Number of patients(%)
**Comorbidities**	Yes	138 (91.4)
	No	13 (8.6)
		
**No of comorbidities**	0	13 (8.6)
	1	71 (47)
	2	54 (35.8)
	3	9 (6)
	≥ 4	4 (2.6)
		
**Medical conditions**		
Diabetes mellitus		46 (30.5)
Hypertension		128 (84.8)
Gouty arthritis		7 (4.6)
Bronchial asthma		6 (4)
Other illnesses		34 (22.5)
**Duration of knee pain**	< 1 y	23 (15.2)
	1 - 5 y	87 (57.6)
	6 - 10 y	40 (26.5)
	> 10 y	1 (0.7)
**Hospitalization for knee pain for the past 1 year **	Yes	8 (5.3)
	No	143 (94.7)
		
**Body Mass Index (BMI)**	18.5 - 24.9 (Normal)	32 (21.2)
	25.0 - 29.9 (Overweight)	76 (50.3)
	> 30.0 (Obese)	43 (28.5)

The profile of SF-36 quality of life dimensions scores are shown in Table 3. A wide range of scores were reported for all dimensions. A full range of scores (0-100) was observed on two of the dimensions: role limitations due to role-physical (RP) and role-emotional (RE). Although the overall mean scores were above 50.00 in all domains, domains related to the physical health status showed relatively lower score as compared to mental health component.

**Table 3:** Overall scores of SF-36 quality of life dimensions

	Physical Health Status				Mental Health Status			
	**Physical Functioning**	**Role-Physical**	**Bodily Pain**	**General Health**	**Vitality**	**Social Functioning**	**Role-Emotional**	**Mental Health**
	**(PF)**	**(RP)**	**(BP)**	**(GH)**	**(VT)**	**(SF)**	**(RE)**	**(MH)**

Mean	51.88	67.54	56.01	53.29	77.84	93.62	84.10	84.95
								
Standard deviation (SD)	24.11	46.16	18.30	17.17	16.33	15.06	36.27	14.79
								
Minimum	5.00	0.00	22.00	5.00	25.00	37.50	0.00	20.00
								
Maximum	100.00	100.00	100.00	100.00	100.00	100.00	100.00	100.00

* Scores range from 0 to 100 with higher scores indicating better functioning.

There was a significant negative correlation between physical functioning (PF) and age (r_s _= -0.337, p < 0.0005) and there were better scores for males in most of the QOL dimensions, especially in the PF domain (p = 0.03). QOL scores were also better pertaining to mental health component for patients with no formal education, where significant differences were found in the vitality (VT) and RE with p = 0.017 and p = 0.007 respectively. There was no significant difference in the marital and employment status.

Patients with co-morbidities scored lower than those without in most of the QOL domains and a significant difference was seen in the social functioning (SF) domain (p = 0.001). The association with other medical characteristics showed negative correlation between duration of knee pain and all the QOL domains (except RE) with significant negative correlation was found in the RP domain (r_s _= -0.287, p < 0.005). There were lower QOL scores for patients with higher BMI except in SF, and significant difference was found in the bodily pain (BP) domain (p = 0.044). A post-hoc analysis showed the significant difference was between normal BMI and obese patient (p = 0.013).

Factors which were significant in the bivariate analysis were then re-analyzed using multiple linear regression analysis. The association between PF scores with age and gender remained significant. Similar results were seen between VT and RE scores with education levels.

## Discussions

The mean age of respondents in our study and the range were similar to a study done previously on mainly Chinese descendents [11]. Majority of our respondents had hypertension followed by diabetes (30.5%). This finding reflected the prevalence of these two diseases among the elderly in Malaysia. A study conducted by the Public Health Department, Universiti Kebangsaan Malaysia reported that the prevalence of hypertension in Hulu Langat, Selangor, Malaysia population increased from 5% among patients between 45-54 years old to 11.6% in those aged 55 - 64 years old. Similar finding was reported in diabetes, 9% rising to 15.8% in older age group [15].

Domains related to the physical health status show relatively lower score as compared to mental health component. The lower score in physical health component compared to the mental component were consistent with other studies [10,11], although they had used different instruments (COOP/WONCA charts and Sickness Impact Profile). One study used the Sickness Impact Profile (SIP) to assess patients in 40 family practices in Netherlands [10]. The other study from Hong Kong, assessed 760 adult Chinese patients of a family medicine clinic with each subject answered the COOP/WONCA charts and a standard questionnaire on demographic and morbidity data [11]. The authors consistently showed that people with OA of the knee had poor quality of life pertaining to the physical activities and overall health compared to the general population.

**Figure 1:**
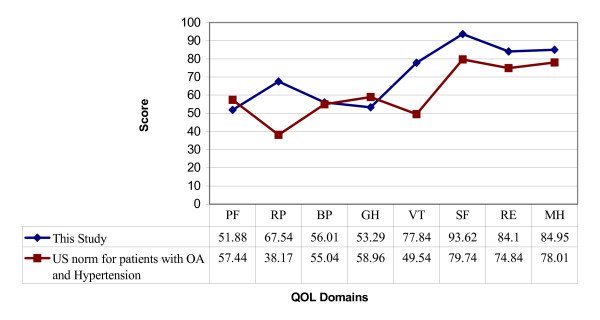
**Comparison between overall mean SF-36 quality of life (QOL) dimension with the US norm for patients with Osteoarthritis and Hypertension**. PF = Physical functioning, RP = Role limitations due to physical health, BP = Bodily pain, GH = General health perceptions, VT = Vitality, SF = Social functioning, RE = Role limitations due to emotional problems, MH = General mental health.

As there was no Malaysian study on QOL of patients with knee OA, a US norm for patients with osteoarthritis and hypertension was used for comparison (Figure 1). This norm was provided in the SF-36 interpretation manual [16]. It represented a quality of life of the population age 55 years and above in US with these two medical conditions. While the physical health component showed virtually identical scores except for RP, the population in our study scored better in the mental health component. A large deficit in RP and VT were seen where the US norm scored much lower compared to the population in this study. In other words, the US population with OA perceived to have more problems with work or other daily activities as a result of physical health and felt tired and worn-out most of the time. This could be due to the difference in coping mechanism, cultural as well as socio-demographic background between the two populations as demonstrated in a few QOL studies amongst Asian population [11,17].

A relatively higher score in the mental component in our study showed that mental health was less affected by knee OA. This could be due to better coping mechanism and adaptation to this chronic disease. Affleck et al [18] studied the coping styles and mood changes in patient with knee OA and rheumatoid arthritis (RA). They found that patients with knee OA used various coping mechanism and resulted in less pain and better mood as compared to patients with RA. Furthermore OA might be considered as normal ageing process, hence it was easily accepted. The affective and cognitive meaning of information that initially was experienced as threatening may be changed to make the present situation more acceptable. Thus patients may have adjusted their expectations for their health or activities downward to the level at which they can function [19].

In our study, age was found to be a predictor for declining of PF. A significant negative correlation was found between age and PF (p < 0.0005), even after adjustment for other confounders. This finding was contrary to previous studies [17,20] where the authors reported an insignificant negative correlation with age. One study looked at factors that were associated with physical functioning in symptomatic knee OA and the other analyzed factors influencing physical function in patients with knee and hip OA attending outpatient clinics.

Female respondents with knee OA were found to have lower scores in most of the QOL dimensions with a significant difference seen in physical functioning (p = 0.03) even after adjusting for other factors (p = 0.024). This is similar to other studies [20,21] and because of the higher prevalence of knee OA in women, this finding is of particular importance to the overall management of patients with knee OA. It has also been shown that being female and having joint stiffness were significant independent predictors of total patient expenditures related to OA [22].

Background education was found to be an important factor associated with VT and RE domains after controlling for confounders. Patient with higher education have lower scores in these two domains. The mechanism by which education influenced mental health is unclear but this could be due to a higher expectation among patients with higher education group. Although there were studies reported that more years of education were associated with better physical function, none of the studies reviewed reported any association between education levels with mental health [11,17,20,23]. Education was also found to have a significant association in PF but disappeared after adjustment. However a small percentage of respondents with higher education in this study limited the generalizability of the results. There was no significance association found when comparing QOL with different employment and marital status which was in agreement with the finding of other studies [17,24].

Despite high prevalence of co-morbidity among respondents in this study, there was no significant association between the presence of co-morbidity and QOL dimensions except for social functioning domain but the latter disappeared after adjustment for confounders. This is in agreement with a previous study [17] that showed co-morbidity had no association with pain or physical functioning.

Respondents with BMI ≥ 30 have lower QOL score in all domains except SF. A significant difference was found in the bodily pain domain even after adjustment for confounders (p = 0.044, p = 0.008 respectively). The association between BMI and the risk of developing knee OA was demonstrable in various other studies too [25-27]. However its relation to pain is less certain. The study by Creamer et al [28] reported a strong correlation between BMI and pain severity. Using WOMAC questionnaire they found that higher BMI was associated with pain on walking, climbing stairs and standing that suggest a direct mechanical role in pain production. Overweight and obesity either lead to an excessive load increase or a medial displacement of the resultant force, depending on the strength of the counteracting lateral muscles. In combination with the observed age-dependent reduction in quadriceps strength and weakening of the lateral muscular tension band, the latter effect could lead to an increased susceptibility of the knee joint [29]. Weight loss has been shown to reduce the incidence of knee OA in a cohort study and is one of the most important preventable risk factors for the knee OA [30].

They were a few limitations in our studies. Chinese and Indian were under represented because of the language difficulty. A validated translation in both languages should be developed in the future to overcome this problem. Small number of male subject limited further extrapolation of the results. Almost all of the study subjects were elderly and therefore some information might have been erroneous due to poor recall.

## Conclusions

This study has shown that patient with knee OA attending primary care have relatively poor quality of life pertaining to the physical health components but there was less impact on the mental health of the patients. Female gender and older age are two important predictors of poor PF in patient with knee OA and patients with higher BMI suffered more pain.

The similarities and differences when compared to other studies on HRQOL of knee OA elsewhere can be attributed to many factors including differences in the utilization of study instrument, selection, definition and size of sample, and the inherent cultural differences that exist between countries. We need a large scale community-based study using a validated multi-languages questionnaire to understand the issues in other ethnic groups and finally, within our setting, primary care doctors should stress on the importance of weight reduction as part of the pain management in knee OA pain.

This study showed that family physicians should try to improve the physical health of patients with knee OA, with particular care for elderly and female patients, and to help relieve the pain in patients with higher BMI.

## Lists of Abbreviations

BMI: Body mass index; BP: Bodily pain; COOP/WONCA: Dartmouth Primary Care Cooperative Information Project/World Organization of National Colleges; Academies, and Academic Associations of General Practice/Family Physicians; HRQOL: Health related quality of life; OA: Osteoarthritis; PF: Physical health; QOL: Quality of life; RA: Rheumatoid arthritis; RE: Role-emotion; RP: Role-physical; SF: Social functioning; SF-36: 36-item short form; SIP: Sickness impact profile; VP: Vitality function; WOMAC: Western Ontario and McMaster Osteoarthritis Index.

## Competing interests

The authors declare that they have no competing interests.

## Authors' contributions

AAB and SYAK participated in the design of the study and performed the statistical analysis. MHH and MFAR involved in the drafting and revising of the manuscript and re-analyzing the data. ZFZ conceived of the study, and participated in its design and coordination and helped to draft the manuscript. All authors read and approved the final manuscript.
